# Psychometric Testing of an Arabic Version of the Attitude Toward Skin-to-Skin Contact Instrument among Women in Saudi Arabia: A Cross-Cultural Validation Study

**DOI:** 10.3390/nursrep14040215

**Published:** 2024-10-11

**Authors:** Abeer Hawsawi, Ritin Fernandez, Maria Mackay, Ibrahim Alananzeh, Abbas Al Mutair

**Affiliations:** 1 College of Nursing, King Saud University, Riyadh 12372, Saudi Arabia; 2 School of Nursing and Midwifery, The University of Newcastle, Newcastle, NSW 2258, Australia; ritin.fernandez@newcastle.edu.au; 3 School of Nursing and Indigenous Health Discipline, University of Wollongong, Wollongong, NSW 2522, Australia; mmackay@uow.edu.au; 4 School of Nursing, The University of Wollongong in Dubai, Dubai P.O. Box 20183, United Arab Emirates; ibrahimalananzeh@uowdubai.ac.ae; 5 School of Nursing, University of Wollongong, Wollongong, NSW 2522, Australia; abbas.almutair@almoosahospital.com.sa

**Keywords:** nursing, skin-to-skin contact, attitude, health and well-being, psychometric testing

## Abstract

Background/Objectives: The World Health Organization recommends skin-to-skin contact immediately after birth, yet the practice rate remains low in Saudi Arabia, impacting the health and well-being of mother–baby dyads. No previous studies have explored Saudi women’s attitudes toward skin-to-skin contact, a critical factor in developing strategies to increase its adoption. This study aimed to develop and evaluate an instrument to assess attitudes toward skin-to-skin contact among women in Saudi Arabia. Methods: An instrument was developed by modifying the validated “Mother–Newborn Skin-to-Skin Contact Questionnaire”. Psychometric testing was conducted to validate the instrument through a cross-cultural survey involving 383 participants recruited from two hospitals in Saudi Arabia using a convenience sampling method. The Kaiser–Meyer–Olkin measure of sampling adequacy was 0.885, indicating that the sample size was suitable for performing exploratory factor analysis. Results: The overall Cronbach’s alpha value was 0.85, reflecting adequate internal consistency of the questionnaire. The criteria of the two-factor confirmatory factor analysis were also met. The majority of women (85.6%) demonstrated a positive attitude towards skin-to-skin contact. A positive correlation was observed between higher educational levels and the total attitude score (r = 0.161, *p* = 0.002). Conclusions: The developed questionnaire is a reliable tool for measuring attitudes towards skin-to-skin contact among women in Saudi Arabia. The findings highlight the importance of educational interventions to improve the uptake of this practice.

## 1. Introduction

The World Health Organization (WHO) recommends placing a newly born baby on the mother’s bare chest just after birth for a minimum of one hour to allow skin-to-skin contact (SSC) [1,2]. This is a crucial procedure as it allows the baby to feel more relaxed and calm, regulating the body’s temperature, breathing, and heart rate and helping the baby adapt to the external environment [3]. During this time, the baby may exhibit species-specific innate behaviours, including smelling, licking, finding, and suckling on the nipple. The early initiation of breastfeeding exposes the baby to colostrum and allows a longer duration of exclusive breastfeeding. In addition, the baby develops a stronger bond with the mother [4]. Furthermore, the SSC practice not only benefits the baby but also the mother in helping with the expulsion of the placenta, as well as preventing haemorrhage, and the mother feels satisfied, happy, and less anxious [5,6]. Therefore, the implementation of SSC is important for the good health and well-being of both mothers and babies, which is in line with the WHO Sustainable Development Goal, SDG 3 [7]. Despite the multiple benefits of SSC to mothers and babies, the uptake is low in many countries, including Saudi Arabia.

The SSC practice rate varies from country to country. The highest rate recorded was 98% in Croatia, followed by 97% in Argentina, while the lowest rate was recorded as 1% in Tanzania [8]. In a recent systematic review, the rate of SSC practice was recorded as 11.11% in Saudi Arabia [9]. There are various barriers that affect SSC uptake in Arab countries, such as lack of appropriate midwifery guidelines, education and training among mothers and nursing staff, beliefs, and cultural practices [9]. The antenatal education in Saudi Arabia normally covers topics such as nutrition, breastfeeding, and postnatal care [10]. However, there is less emphasis on the importance of skin-to-skin contact (SSC) practice. The disparities in SSC practice between countries can partly be explained by the cultural differences that have an influence on the attitude towards SSC [11]. It is a common practice to take the baby away from the mother immediately after birth to clean and carry out postnatal procedures, while the mother is resting [11]. Furthermore, fathers cannot help mothers with skin-to-skin contact as they are not allowed in the birth room during childbirth [12]. Although Saudi women reported that they did not feel that SSC was against the norms of modesty in Saudi culture, the low uptake of SSC could not be explained. Very few studies have been carried out in Saudi Arabia regarding SSC, and none could be found regarding attitude of SSC among Saudi mothers [9]. However, a recent study carried out in Al-Ahsa, Saudi Arabia, demonstrated that nursing staff have reasonable knowledge, attitude, and implementation skills of SSC [13]. This highlights the importance of exploring mothers’ attitudes in order to develop an effective intervention. Understanding these attitudes may reveal the cultural, educational, and systemic barriers that may hinder the practice of SSC.

Attitudes can predict behaviour, and although they can evolve with time, there are some types of attitudes that are deeply entrenched and cannot be influenced or altered [14]. Given that the uptake of skin-to-skin contact (SSC) is low in Saudi Arabia, there is a need to assess attitude of SSC among mothers to help find a solution to the problem. This will give insight into the awareness and understanding of the importance of SSC among women in Saudi Arabia and will be useful in guiding the type of intervention that will be required. Nevertheless, one of the studies in Saudi Arabia reported mothers’ perceptions about immediate SSC, where the majority of women reported favourable perceptions about SSC and disagreed that the practice was against their modesty and culture [11]. Before embarking on the development of an intervention to improve the SSC practice, it is important to assess mothers’ attitudes as there is a clear distinction between cultural norms, perceptions, and attitudes, and all three may have an impact on SSC practice. While perception refers to a mother’s understanding of behaviour (SSC practice) in relation to cultural norms (the “shoulds” and the “should nots”), attitude is the predisposed feeling, which could be positive or negative towards the behaviour [15]. The overall attitude can be influenced by both cultural norms and perceptions, and if mothers have misconceptions or disbeliefs about the benefits of the SSC practice, then the proposed intervention may become ineffective. Therefore, there is a need to assess the attitude towards SSC in Saudi Arabia to understand the root cause of the low practice and guide the development and implementation of culturally sensitive and effective interventions.

The measurement of attitude through self-reports in the form of questionnaires is one of the several approaches that are currently employed [14]. However, the main barrier to assessing the attitude of SSC among Saudi women is the lack of a validated questionnaire. The author found a reliable questionnaire, the “Mother–Newborn Skin-to-Skin Contact Questionnaire (MSSCQ)”, which was developed by Nahidi and colleagues, to assess factors associated with skin-to-skin immediately after birth in Iran [16]. The MSSCQ comprised 11 items in the “Predisposing factors” section that were used to assess the attitude towards SSC among the nursing staff [16]. The scoring was based on a 5-point Likert scale ranging from “Strongly Disagree” (Score 1) to “Strongly Agree” (Score 5). Validation of the MSSCQ demonstrated that Cronbach’s α coefficient ranged from 0.84 to 0.89, indicating satisfactory internal consistency. Furthermore, the Intraclass Correlation Coefficient (ICC) also was satisfactory, which confirmed the reliability of the MSSCQ. Therefore, the MSSCQ was deemed to be a reliable tool that could be adapted to develop a new questionnaire. To date, no study has been carried out in Saudi Arabia that reported the attitude towards SSC. This paper reports on the development and validation of a questionnaire to assess the attitude towards SSC among women in Saudi Arabia.

## 2. Materials and Methods

### 2.1. Questionnaire Development 

This study was carried out in two phases. In the first phase, the Skin-to-Skin Contact Attitude Questionnaire (SSCQ-Attitude) instrument was developed by modifying the MSSCQ and adapting it to assess the attitude towards SSC. The MSSCQ has 11 items that assess the attitude of healthcare providers towards SSC. Items 3, 8, 9, and 10 from the “Predisposing factors” section of the MSSCQ were adapted for the attitude section of the SSCQ-Attitude. One of the items was reversed as follows: Item 10, “Skin contact reduces mother’s stress” was reversed to “I believe skin-to-skin increases mother’s stress”. The modification of the attitude items focussed on assessing the attitudes among mothers rather than health care providers as it was originally designed [16]. The questionnaire adopted the 5-point Likert scale as it includes a wider range of respondent options to decrease the central tendency error and response bias. This offers better validity and discriminating power by capturing the accurate opinions of respondents [17]. While some of the questions were adapted from the MSSCQ, the remaining questions of the SSCQ-Attitude were developed by the expert committee based on a review of the literature to generate a total of 10 items to assess attitudes towards SSC among women in Saudi Arabia. The questionnaire was then translated into Arabic by three Arabic/English-speaking bilingual experts, ensuring that the clarity and meaning were retained. A forward–backward translation process was used to ensure the functional equivalence between the English and Arabic versions of the tool [18]. The content validity was established by a panel of experts, including three nurse educators with extensive experience in obstetrics and maternity and two university scholars who were bilingual experts. They were asked to check the relevance of the questions and whether any modification was required. A pilot study among 25 respondents, including healthcare providers and university researchers was also carried out. Based on the findings of the expert panel and the pilot study, the content validity of the SSCQ-Attitude was found to be satisfactory, and no change was made to the items as the clarity and relevance of the questionnaire were acceptable with no significant issues reported. The items of the SSCQ-Attitude, which was available both in English and Arabic to assess attitude, were finally confirmed.

In the second phase, the SSCQ-Attitude was validated through a prospective cross-cultural survey among pregnant women recruited using convenience sampling from two hospitals in Saudi Arabia. Reliability, which is a measure of the reproducibility of a questionnaire, was assessed using Cronbach’s α test [19,20].

### 2.2. Settings and Participants

The study settings were two large tertiary hospitals in Riyadh, Saudi Arabia. The two Hospitals are multi-disciplinary facilities providing tertiary care to patients in a range of areas, including general medicine, surgical, and specialised services such as obstetrics and maternity. Participants were included if they were (1) healthy pregnant women in their third trimester attending the obstetrics and gynaecology clinic of the participating hospitals and (2) able to read and write in English or Arabic. Participants were excluded if they were (1) women under 18 years old, because of legal and social circumstances that may affect their autonomy in decision-making regarding SSC, and (2) high-risk pregnancies to ensure the safety of both mothers and neonates and reduce variability of the findings.

### 2.3. Participants Recruitment

This study received ethical approval both from the University as well as the study settings. All eligible pregnant women attending the obstetrics and gynaecology clinic were informed of this study by the nurses, who invited them to participate. Participant Information sheets with a barcode link to the electronic questionnaire were distributed to all pregnant women who were willing to participate in this study. They were informed that they could opt out of this study at any time. The participants were asked to complete the online questionnaire on Qualtrics. Completion of the online questionnaire was considered as confirmation of informed consent.

### 2.4. Data Analysis

The data from the online questionnaire on Qualtrics were downloaded into Excel (Microsoft 365) and analysed using SPSS version 29. The scores were reversed where appropriate. Means, standard deviations, and frequency distributions were used to describe the demographics of the participants. To check for sample adequacy, the Kaiser–Meyer–Olkin (KMO) index was calculated, and the distribution of the “participant” responses was evaluated using “Bartlett’s test of sphericity” [21]. Exploratory factor analysis (EFA) was used to determine the dimensionality and interrelationships between the variables in a set of data [21]. Principal Components Analysis (PCA) with Varimax Rotation was applied to perform the exploratory factor analysis. Kaiser’s (Eigenvalue) Criterion and the Scree Test were used to extract the initial unrotated components. Using this approach, only factors with eigenvalues greater than one were retained. However, those items that generated ≥ 0.50 were also retained on the respective factor [22,23]. Furthermore, according to face validity and interpretability, cross-loading items were retained only if they were related to each other in a meaningful manner [24].

Confirmatory factor analysis (CFA) was subsequently conducted using AMOS 24.0 software to assess the construct validity of the two-factor model and to further explore its dimensional structure. Confirmatory factor analysis is used to test the hypothesis of a specific relationship between variables and latent factors. It is also used to evaluate a model’s ability to express the actual data set and compare several models. In this study, the criteria for confirmatory factor analysis were verified through a value of the “significance level for the chi-square test” of less than 0.05 and (CMIN/DF) less than 5, the goodness-of-fit index (GFI), the normed fit index (NFI), the comparative fit index (CFI), the relative fit index (RFI), the incremental fit index (IFI), and the Tucker and Lewis index (TLI). These indices must exceed 0.9 to be accepted within the verification of model fit, and the root mean square error of approximation (RMSEA), and standardized root mean square residual (SRMR) must be less than 0.10 [25].

Cronbach’s alpha test was performed for each item of the scale as well as the total scale to assess the internal reliability of the questionnaire [21]. A Cronbach’s alpha of 0.70 to 0.80 was considered acceptable, and >0.8 was considered very good [23].

Construct validity is used to determine if a construct measures what it is intended to measure, which helps to interpret the findings [18]. This can be achieved by finding the correlation with other variables. The known-group technique was used to compare the SSCQ-Attitude scores between the identified groups [26]. Pearson correlation analysis was performed to determine a relationship between the attitude scores and demographic characteristics such as age, educational level, employment status, and number of children. A correlation matrix was then used to examine the patterns of associations between the variables. A correlation coefficient of 0.1 was considered small, 0.3 was considered moderate, and 0.5 was considered large [18].

To assess attitude, a score of 1 to 5 was given based on the 5-point Likert scale for attitude, with the highest score allocated to a highly positive attitude, and scores were reversed for the reverse items. The scores were collated and expressed as mean and standard deviation. For each participant, the minimum achievable score was 10 and the maximum achievable score was 50, and a participant with a total score of >30 was deemed to hold a positive attitude towards SSC.

## 3. Results

### 3.1. Instrument Modification, Content Validity, and Pilot Testing

The SSCQ-Attitude was developed by adapting some items from the MSSCQ to assess the attitudes among Saudi women towards SSC. The MSSCQ has 11 items that assess the attitudes of healthcare providers towards SSC. Some items from the “Predisposing factors” section of the MSSCQ were adapted for the attitude section of the SSCQ-Attitude. As stated previously, Item 10, “Skin contact reduces mother’s stress”, was reversed to “I believe skin-to-skin increases mother’s stress”. The instrument consists of 10 items that assess attitudes towards SSC with a five-point Likert scale from “strongly disagree” to “strongly agree”. The scores for attitude range from a minimum of 10 to a maximum of 50, with an average of 30. For content validity, the expert panel provided positive feedback about the questionnaire items for relevance, clarity, and comprehensiveness, and some minor changes were made regarding the wording of specific items. Based on the findings of the expert panel and the pilot study involving 25 respondents, the content validity of the SSCQ-Attitude was found to be satisfactory, and no change was made to the items of the SSCQ-Attitude that were used in the second phase.

### 3.2. Response Rate and Demographics

A total of 383 women visiting the two Saudi hospitals from October 2022 to February 2023 completed the survey, and all data were included in the final analysis. The mean age was 31.7 years (+/− 5.2), and the majority (74.7%) of the women were unemployed, as shown in Table 1.

### 3.3. Validation of the SSCQ-Attitude 

#### 3.3.1. Exploratory Factor Analysis (EFA)

**Figure 1:**
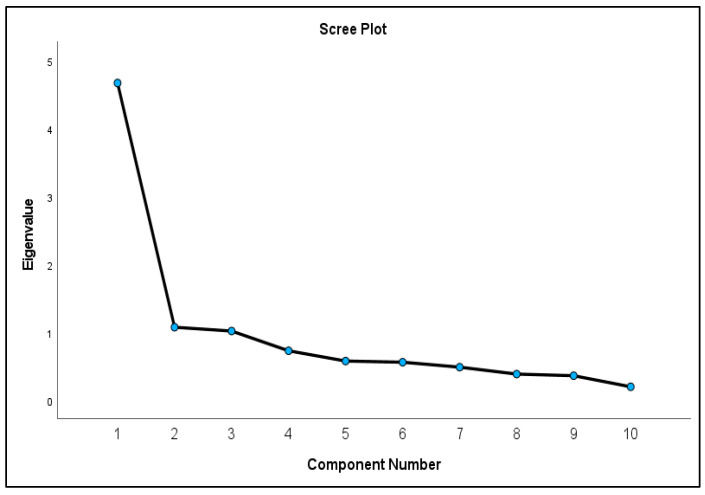
Scree plot of the factor eigenvalues.

Before undertaking the factor analysis, the Kaiser–Meyer–Olkin measure of sampling adequacy was performed and found to be 0.885, which is above the recommended value of 0.60. Furthermore, Bartlett’s test of sphericity reached statistical significance (chi-square = 1603.64, *p* < 0.001) for the SSCQ-Attitude, which indicated that the variables are correlated. These findings indicated that the data were suitable for factor analysis. Visual inspection of the scree plot of the SSCQ-Attitude identified three factors with eigenvalues greater than 1, accounting for 68% of the total variance, as shown in Figure 1.

A clear departure from linearity consistent with a three-factor solution was revealed. Communality values were greater than 0.53 for all items, and the factor loadings ranged from 0.512 to 0.967 (Table 2). Only one item, “I believe SSC increases mother’s stress”, loaded on the third factor. However, it was included with the first factor as it fit well with the items about love, care, and the bond between mother and baby. Therefore, only two factors were identified and labelled as “Bond between mother and baby” and “Upholding Cultural values”. The first factor comprises seven items, while the second is composed of three items and the Cronbach’s alpha values were 0.794 and 0.744, respectively. The overall Cronbach’s alpha value was 0.85, which indicates adequate internal consistency; therefore, the SSCQ-Attitude is a reliable tool to measure attitudes among Saudi women.

#### 3.3.2. Confirmatory Factor Analysis

**Figure 2:**
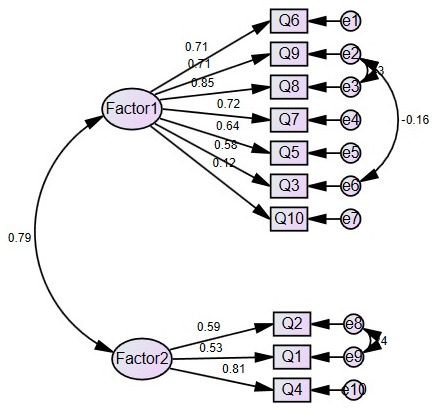
Confirmatory factor analysis for attitude.

Further analysis of the overall goodness of fit indicated that the criteria for confirmatory factor analysis of the dimension (attitude) were met, and the results were (CMIN/DF = 3.513 < 5, Chi_ Square_ sig = 0.000 < 0.05, “GFI = 0.951, CFI = 0.956, TLI = 0.936, NFI = 0.938, RFI = 0.910 > 0.90”, “RMSEA = 0.077, SMRMR = 0.030 < 0.10”). The two-factor confirmatory factor analysis model with factor loadings is shown in Figure 2.

### 3.4. The Reliability of the SSCQ-Attitude

The internal consistency of the SSCQ-Attitude with all ten items was found to be high (α = 0.85, M = 3.80). The Cronbach’s alpha values for the two factors “Bond between mother and baby” and “Upholding Cultural values” were 0.794 and 0.744, respectively.

### 3.5. Attitudes towards SSC

In the SSCQ-Attitude, the average achievable score for attitude is 30, with a minimum achievable score of 10 and maximum achievable score of 50. The minimum score was 14, the maximum was 50, and the mean score for attitude was 37.97 (SD 5.53). A total of 328 participants (85.6%) scored greater than 30, demonstrating a good attitude towards SSC. Pearson correlation was performed to investigate any relationship between age, employment status, number of children, educational categories, and attitude. No correlation was found except for the educational level, where a positive correlation was found with factor 1, “Bond between mother and baby” (r = 0.216, *p* < 0.001), and the total attitude score (r = 0.161, *p* = 0.002) (Table 3).

## 4. Discussion

A scarcity of comprehensive research concerning attitudes related to SSC within Saudi hospitals is evident. Concurrently, an absence of validated instruments designed for assessing the attitude towards SSC among Saudi women is also apparent. These gaps reinforced the critical need for focused investigation and the development of appropriate assessment tools in this context. Therefore, this study aimed to adapt the MSSCQ [16] to develop the SSCQ-Attitude with the help of university researchers in Australia and translate it into Arabic to assess the attitude towards SSC among women in Saudi Arabia. The content validity was enhanced following a pilot study involving 25 Saudi women followed by an online survey. This paper focussed on the validation of the SSCQ-Attitude as well as reported on the attitude towards SSC among women in Saudi Arabia.

Attitude is one of the three most important pillars for health improvement through behavioural change, and all three elements are interdependent [27]. Although a recent study reported that the majority of women in Saudi Arabia held favourable, positive perceptions towards SSC [9,11], no study could be found that reported on attitude towards SSC. Furthermore, most Saudi women could not practice SSC properly [9,11]. This is due a lack of hospital guidelines coupled with the absence of proper SSC training of health care professionals and mothers [28]. Educating and training health care professionals is less effective if mothers are not educated about SSC during antenatal care. A lack of awareness about SSC could explain the current poor SSC practice in Saudi Arabia. Health education for the public is vital for better health outcomes as it improves people’s behaviours [27]. This advocates for an educational intervention in Saudi Arabia to increase the awareness of SSC practice among women and health care providers.

This is the first study reporting Saudi women’s attitudes towards SSC. Around 85.6% *(n* = 328) of Saudi women demonstrated a positive attitude towards SSC. This is well above the figure reported from Southern Ethiopia, where 50.3% of mothers demonstrated a good attitude towards SSC practice [29]. Furthermore, an assessment of the attitudes towards SSC among Saudi women in the two hospitals revealed a weak positive correlation between attitude and educational categories (r = 0.216, *p* < 0.001). Participants with higher educational categories, such as degrees and post-graduates, held more positive attitudes towards SSC. This is in line with several studies where a good correlation was found between attitude and the level of education [30,31,32]. Having better knowledge not only influences attitude but positively affects a change in behaviours, which are required to improve health outcomes. Therefore, an educational intervention tailored according to the needs of women in Saudi Arabia is necessary to improve the health and well-being of mother–bay dyads through SSC practice. As the rate of SSC practice is low in Saudi Arabia, there is an urgent need to improve the uptake of SSC practice through educational intervention [9,33].

### Strengths and Limitations

One of the strengths of this study is that the SSCQ-Attitude is available both in English and Arabic, facilitating understanding by allowing personal preference. Another strength is the ease and convenience offered to participants by making the questionnaire available online. This allowed the participants to fill in the questionnaire at their own pace or save it and return back to the questionnaire to complete the survey. This could explain why the sample size was large enough to achieve proper validation.

This study has several limitations that are worth mentioning. Firstly, convenience sampling was used because randomisation was not possible because of COVID-19 and limited time restrictions. Convenience sampling has its own disadvantages, including the risk of potential biases and lack of generalisability due to regional, socioeconomic, or cultural specificity. This could have had a negative impact on the generalisability of this study findings. Future studies may consider the randomisation of participants during recruitment from various parts of Saudi Arabia to take part in the online survey. Larger multi-centre studies including women in the second trimester will allow a larger sample size with increased diversity. This will enhance the generalisability of the findings to the population of Saudi Arabia. Future studies may also consider the assessment of attitude among healthcare providers as this may provide a more comprehensive view of the SSC practice landscape requiring specific interventions.

## 5. Conclusions

The current study has successfully modified the MSSCQ, translated the questionnaire, and validated the SSCQ-Attitude, which is available both in Arabic and English. The validation findings demonstrated high reliability and validity, which means that the SSCQ-Attitude is a good instrument that can be used to reliably assess the attitude towards SSC among Arab women. In general, most Saudi women were found to hold a positive attitude towards SSC. However, the practice of SSC is very low, affecting the health and well-being of mothers and babies, which necessitates an education intervention to improve the practice of SSC in Saudi Arabia.

## Figures and Tables

**Table 1:** Demographic characteristics of the participants.

	Number (N)	Minimum	Maximum	Mean	SD
Age	383	18	44	31.66	5.19
No. of children	383	0	8	2.16	1.66
			Frequency	Per cent	Cumulative Percentage
Employment status					
Employed			97	25.3	25.3
Unemployed			286	74.7	100.0
Educational level					
High education			25	6.5	6.5
University degree			208	54.3	60.8
High school or less			150	39.2	100.0

**Table 2:** Factor analysis of the SSCQ-Attitude.

Feature	Factor Loading			Communalities (h2)	Mean (SD)
	1	2	3		
Factor 1: Bond between mother and baby (α = 0.794)					
I believe SSC enhances a mother’s love for the baby	0.878	0.152	0.089	0.802	4.06 ± 0.79
I believe SSC calms and relaxes both the mother and baby	0.838	0.185	0.124	0.752	3.94 ± 0.81
I believe SSC creates a sense of security between the mother and baby	0.778	0.144	0.081	0.633	3.87 ± 0.77
I believe SSC establishes an emotional bond between mother and baby	0.714	0.271	−0.022	0.584	3.91 ± 0.79
I believe SSC increases a mother’s confidence in caring for the baby	0.675	0.263	−0.106	0.536	3.69 ± 0.83
I believe SSC is supported by religion	0.512	0.494	−0.191	0.542	3.68 ± 0.85
I believe SSC increases a mother’s stress ^1^	0.061	0.073	0.967	0.945	3.52 ± 0.91
Factor 2: Upholding Cultural values (α = 0.744)					
I believe SSC is culturally appropriate for Saudi women	0.152	0.835	0.154	0.743	3.72 ± 0.95
I would like to know more about skin-to-skin contact (SSC) during my antenatal visits	0.18	0.728	0.022	0.563	3.89 ± 0.92
I believe SSC is supported by family	0.556	0.578	−0.058	0.647	3.70 ± 0.84

^1^ Reverse-coded item.

**Table 3:** Correlation between demographics and attitude.

Variables		1	2	3	4	5	6	7
1	Age	1						
2	Employment status	−0.266 **	1					
3	No. of children	0.408 **	0.021	1				
4	Educational level	−0.111 *	0.331 **	0.224 **	1			
5	Factor 1 (Bond between mother and baby)	0.034	−0.089	−0.083	0.216 **	1		
6	Factor 2 (Upholding Cultural Values)	0.008	−0.037	−0.077	−0.055	0.622 **	1	
7	Total Attitude score	0.025	−0.074	−0.090	0.161 **	0.925 **	0.873 **	1

*. Correlation is significant at the 0.05 level (2-tailed). **. Correlation is significant at the 0.01 level (2-tailed).

## Data Availability

The data supporting the reported results of this study are available upon request. Please contact the corresponding author for access to the data.

## Public Involvement Statement

There was no public involvement in any aspect of this research.

## Guidelines and Standards Statement

This manuscript was drafted against the STROBE (Strengthening the Reporting of Observational Studies in Epidemiology) guidelines for cross-sectional research. A complete list of reporting guidelines can be accessed via the EQUATOR Network at https://www.equator-network.org/. (accessed on 15 June 2024).

## References

[1] Widström A.M., Brimdyr K., Svensson K., Cadwell K., Nissen E. (2019). Skin-to-skin contact the first hour after birth, underlying implications and clinical practice. Acta Paediatr..

[2] World Health Organisation Skin-to-Skin Contact Helps Newborns Breastfeed. https://www.who.int/westernpacific/news-room/feature-stories/item/skin-to-skin-contact-helps-newborns-breastfeed.

[3] Byrom A., Thomson G., Dooris M., Dykes F. (2021). UNICEF UK Baby Friendly Initiative: Providing, receiving and leading infant feeding care in a hospital maternity setting—A critical ethnography. Matern. Child Nutr..

[4] Nyqvist K.H., Anderson G.C., Bergman N., Cattaneo A., Charpak N., Davanzo R., Ewald U., Ibe O., Ludington-Hoe S., Mendoza S. (2010). Towards universal Kangaroo Mother Care: Recommendations and report from the First European conference and Seventh International Workshop on Kangaroo Mother Care: Towards universal Kangaroo Mother Care. Acta Paediatr..

[5] Moore E.R., Anderson G.C., Bergman N., Dowswell T. (2012). Early skin-to-skin contact for mothers and their healthy newborn infants. Cochrane Database Syst. Rev..

[6] Essa R.M., Ismail N. (2015). Effect of early maternal/newborn skin-to-skin contact after birth on the duration of third stage of labor and initiation of breastfeeding. J. Nurs. Educ. Pract..

[7] Sachs J.D., Kroll C., Lafortune G., Fuller G., Woelm F. (2022). Sustainable Development Report 2022.

[8] Abdulghani N., Edvardsson K., Amir L.H. (2018). Worldwide prevalence of mother-infant skin-to-skin contact after vaginal birth: A systematic review. PLoS ONE.

[9] Hawsawi A., Fernandez R., Mackay M., Alananzeh I., Al Mutair A. (2022). Knowledge, Attitudes, Practices, Barriers and Facilitators to Skin-To-Skin Contact among Arabian Mothers and Health Care Providers in Arab Countries: A Systematic Scoping Review. Int. J. Childbirth.

[10] Ministry of Health Saudi Midwifery Clinics Standards. https://www.moh.gov.sa/en/Ministry/MediaCenter/Publications/Pages/The-Saudi?Midwifery-Clinic-Standards.pdf.

[11] Abdulghani N., Cooklin A., Edvardsson K., Amir L.H. (2022). Mothers’ perceptions and experiences of skin-to-skin contact after vaginal birth in Saudi Arabia: A cross-sectional study. Women Birth.

[12] Alharbi A.A., Alodhayani A.A., Aldegether M.S., Batais M.A., Almigbal T.H., Alyousefi N.A. (2018). Attitudes and barriers toward the presence of husbands with their wives in the delivery room during childbirth in Riyadh, Saudi Arabia. J. Fam. Med. Prim. Care.

[13] Almutairi W.M. (2022). Survey of Skin-to-Skin Contact with Obstetrics and Pediatric Nurses. Nurs. Rep..

[14] Brown T., Isbel S., Yu M.-L., Bevitt T. (2020). Measuring Attitudes: Current Practices in Health Professional Education. Clinical Education for the Health Professions: Theory and Practice.

[15] Hudson R.A., Rosen H. (1953). On the definition of attitude: Norms, perceptions, and evaluations. Public Opin. Q..

[16] Nahidi F., Tavafian S.S., Heidarzadeh M., Hajizadeh E., Montazeri A. (2014). The Mother-Newborn Skin-to-Skin Contact Questionnaire (MSSCQ): Development and psychometric evaluation among Iranian midwives. BMC Pregnancy Childbirth.

[17] Joshi A., Kale S., Chandel S., Pal D.K. (2015). Likert scale: Explored and explained. Br. J. Appl. Sci. Technol..

[18] Tsang S., Royse C.F., Terkawi A.S. (2017). Guidelines for developing, translating, and validating a questionnaire in perioperative and pain medicine. Saudi J. Anaesth..

[19] Heale R., Twycross A. (2015). Validity and reliability in quantitative studies. Evid.-Based Nurs..

[20] Tavakol M., Dennick R. (2011). Making sense of Cronbach’s alpha. Int. J. Med. Educ..

[21] Finch W.H. (2020). Exploratory Factor Analysis.

[22] Osborne J.W., Costello A. (2009). Best practices in exploratory factor analysis: Four recommendations for getting the most from your analysis. Pan-Pac. Manag. Rev..

[23] Abd ElHafeez S., Salem M., Silverman H.J. (2022). Reliability and validation of an attitude scale regarding responsible conduct in research. PLoS ONE.

[24] Brown T.A. (2015). Confirmatory Factor Analysis for Applied Research.

[25] Lorenzo-Seva U., Ferrando P.J. (2020). Unrestricted factor analysis of multidimensional test items based on an objectively refined target matrix. Behav. Res. Methods.

[26] LoBiondo-Wood G., Haber J. (2014). Reliability and validity. Nursing Research-ebook: Methods and Critical Appraisal for Evidence-Based Practice.

[27] Badran I.G. (1995). Knowledge, attitude and practice the three pillars of excellence and wisdom: A place in the medical profession. East. Mediterr. Health J..

[28] Alenchery A.J., Thoppil J., Britto C.D., de Onis J.V., Fernandez L., Rao P.S. (2018). Barriers and enablers to skin-to-skin contact at birth in healthy neonates-a qualitative study. BMC Pediatr..

[29] Mose A., Adane D., Abebe H. (2021). Skin-to-Skin Care Practice and Its Associated Factors Among Postpartum Mothers in Gurage Zone, Southern Ethiopia: A Cross-Sectional Study. Pediatr. Health Med. Ther..

[30] Diaz-Quijano F.A., Martínez-Vega R.A., Rodriguez-Morales A.J., Rojas-Calero R.A., Luna-González M.L., Díaz-Quijano R.G. (2018). Association between the level of education and knowledge, attitudes and practices regarding dengue in the Caribbean region of Colombia. BMC Public Health.

[31] Rahman R., Qattan A. (2021). Vision 2030 and Sustainable Development: State Capacity to Revitalize the Healthcare System in Saudi Arabia. INQUIRY J. Health Care Organ. Provis. Financ..

[32] ul Haq N., Hassali M.A., Shafie A.A., Saleem F., Farooqui M., Aljadhey H. (2012). A cross sectional assessment of knowledge, attitude and practice towards Hepatitis B among healthy population of Quetta, Pakistan. BMC Public Health.

[33] Abdulghani N., Amir L.H., Edvardsson K. (2020). Observational study found that skin-to-skin contact was not common after vaginal birth in Saudi Arabia. Acta Paediatr..

